# Discovering the anti-diabetic potential of pomegranate peel metabolites by examining molecular interplay with the thioredoxin-interacting protein

**DOI:** 10.3389/fmed.2023.1322450

**Published:** 2024-09-10

**Authors:** Tahira Aslam, Amina Arif, Shafia Arshad, Fatima Muccee, Khalil Ahmad, Muhammad Omer Iqbal, Umair Khalil, Suhail Razak, Tayyaba Afsar, Ali Almajwal, Huma Shafique, Maryam Zain

**Affiliations:** ^1^Department of Basic and Applied Chemistry, Faculty of Science and Technology, University of Central Punjab, Lahore, Pakistan; ^2^University College of Conventional Medicine, Faculty of Medicine and allied Health Sciences, The Islamia University of Bahawalpur, Bahawalpur, Pakistan; ^3^School of Biochemistry and Biotechnology, University of Punjab, Lahore, Pakistan; ^4^Key Laboratory of Marine Drugs, the Ministry of Education, School of Medicine and Pharmacy, Ocean University of China, Qingdao, China; ^5^Department of Community Health Sciences, College of Applied Medical Sciences, King Saud University, Riyadh, Saudi Arabia; ^6^Institute of Cellular Medicine, Newcastle University Medical School, Newcastle University, Newcastle upon Tyne, United Kingdom; ^7^Department of Biochemistry and Biotechnology, The Women University Multan, Multan, Pakistan

**Keywords:** *Punica granatum*, TXNIP, diabetes, *Staphylococcus viridans*, phytochemicals, biological activities

## Abstract

**Introduction:**

Medicinal plants like Punica granatum (pomegranate) have traditional uses against diabetes, inflammation and other diseases. The study was initiated to get an insight into the interaction tendency of *P. granatum* derived compounds with diabetes associated human thioredoxin-interacting protein (TXNIP). High glucose in diabetes induces production of TXNIP resulting in β-cells apoptosis. Its inhibition might reduce the diabetes incidence.

**Methods:**

To elucidate the therapeutic potential of *P. granatum* peel against diabetes through GC-MS based identification of extracted compounds followed by application of computational algorithms. *P. granatum* peel extracts were screened for antioxidant, anti-inflammatory, anti-diabetic, antimicrobial and wound healing properties. Phytochemical and GC-MS based analysis were performed to identify the bioactive compounds. Molecular docking analysis was performed by Auto Dock Vina to predict the binding tendency of *P. granatum* derived compounds with TXNIP.

**Results and Discussion:**

The peel exhibited antioxidant, anti-inflammatory and anti-diabetic activities, which were attributed to phytochemicals like phenols, tannins and steroids. GC-MS analysis identified 3,5-octadien-2-one, 1H-pyrrole -2,5-dione, Beta-D-lyxofuranoside, 5-O-(beta-D-lyxofuranosyl)-decyl, diethyl phthalate, 9-octadecenoic acid (Z)-, methyl ester, hexadecanoic acid, methyl ester, n-hexadecanoic acid, tetradecane, 2,6,10-trimethyl, bis (2-ethylhexyl) phthalate, decane, 3,8-dimethyl, 9-octadecenoic acid (Z)-, methyl ester and bis (2-ethylhexyl) phthalate in *P. granatum* peel extracts. Docking analysis revealed high binding affinities of bis (2-ethylhexyl) phthalate and 9-octadecenoic acid with TXNIP i.e., –4.5 and –5.0 kcal/mol, respectively, reflecting these compounds as potent antidiabetic agents. This study validates the traditional uses of *P. granatum* peel and demonstrates how computational approaches can uncover pharmacologically active phytochemicals. The results suggest *P. granatum* peel is a promising source of novel therapeutics against diabetes, inflammation, and oxidation. Further studies on the optimization of identified ligands are warranted.

## Graphical Abstract

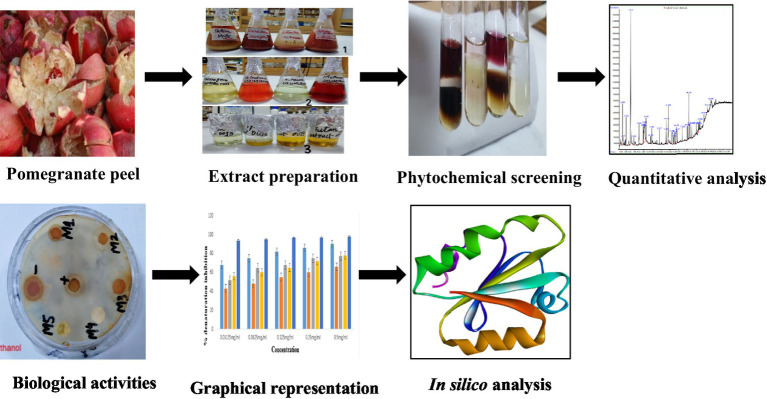

## Introduction

**Figure 1:**
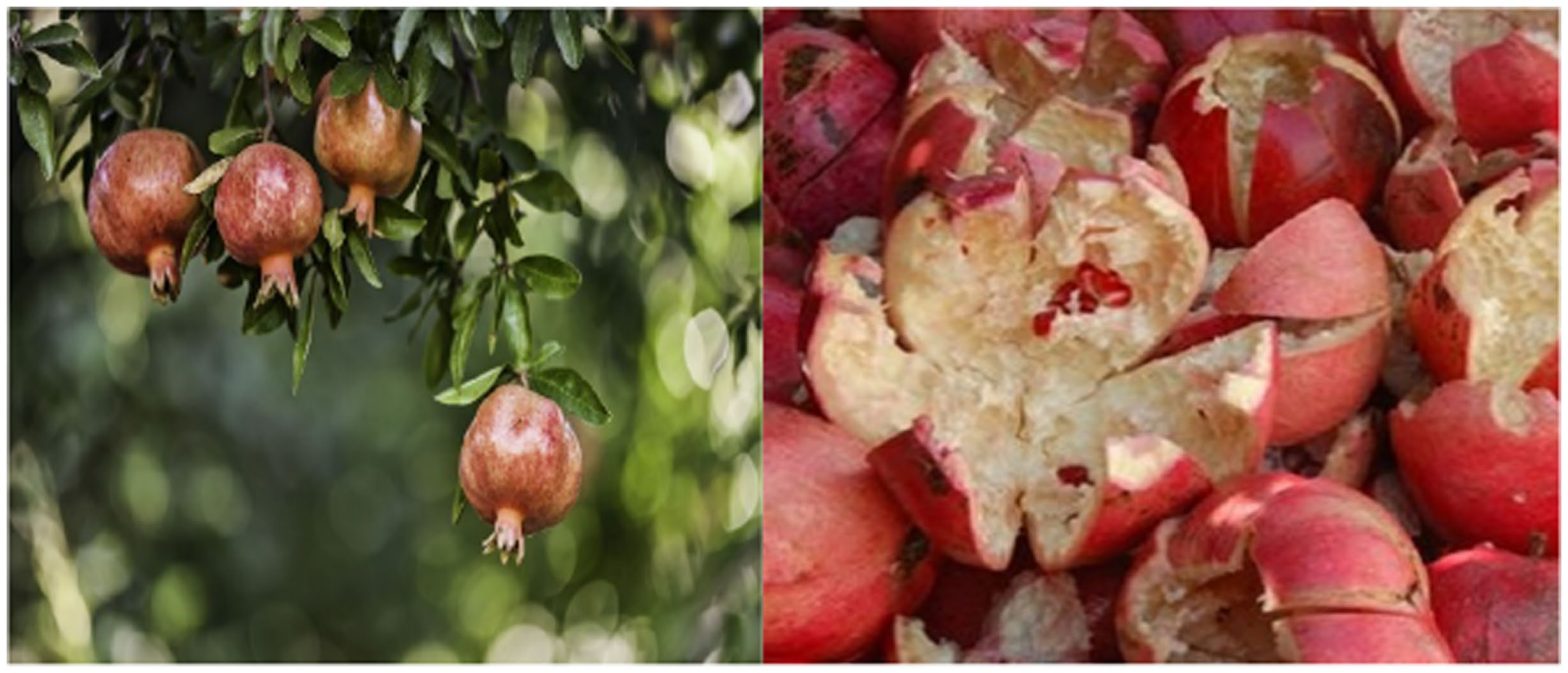
*Punica gramatum* plant and peel.

Plants have been used for centuries in traditional medicines (1). Different diseases can be treated by using traditional techniques. The word pomegranate is derived from “granatus” and “ponus” which are Latin words. The scientific classification of the plant is presented in Table 1. Approximately 1,500,000 tonnes of pomegranate are produced worldwide. Mediterranean-native pomegranate (*Punica granatum* L.) is used frequently in traditional medicines in several countries, especially throughout the Indian subcontinent. Pomegranate contains approximately 60% peel in its fruit (3). The structure of the plant and peel is shown in Figure 1. It has the ability to perform antifungal, antibacterial, antioxidant, immune-modulatory, anti-atherosclerotic, and anti-bacterial activity (4), as well as having wound healing capacity; cardiovascular diseases, gum bleeds, diabetes, male infertility, eye disorders, infant brain ischemia, and throat issues can be treated with pomegranate plant. It also contains various phytochemical properties. Punicalins, ellagic acid, punicalagins, gallic acid, and gallotannis are the phytochemicals present in the peel. According to reports, the fruit’s peel and juice contain 10–50 mg/100 g and 1–2.38 mg/100 mL of ellagic acid. Numerous bioactive compounds and secondary metabolites such as flavonoids, tannins, phenolic, fibers, vitamins, and minerals, rich in polyphenols and tannins, are present in the peel (1). The non-edible parts including flowers, barks, peel, leaves, and buds play significant roles in the nutritional characteristics, in addition to the fruit’s edible part. Compared to the fruit’s edible portion, the peel is usually known as waste, but it contains biologically active compounds rich in nutrients (5).

**Table 1:** Scientific classification of pomegranate (2).

Kingdom	Plantae
Division	Magnoliophyta
Class	Magnoliopsida
Subclass	Rosidae
Order	Myrtales
Family	Lythraceae
Genus	Punica
Species	*P. granatum*

Table 1 shows the scientific classification of pomegranate.

### Biological compounds of plant

Pomegranate is rich in bioactive compounds, and they have demonstrated a variety of therapeutic benefits. High concentrations of hydrolyzed ellagitannins known as “pomegranate ellagitannins,” which include pedunculgins, punicalins, and punicalagins, are present in pomegranate, especially in the peel (6). Hexahydroxy diphenic acid and polyol such as glucose or quinin acid are combined to form ellagitannins and are known as esters. The red part of the fruit contains flavonoids (7), which include quercetin, kaempferol, and luteolin. Ellagitannins have hydroxybenzoic acids such as ellagic acid, gallic acid, and ellagic acid glycosides. Anthocyanidins like delphinidin, cyaniding, and pelargonidin, as well as anthocyanins, the pigment responsible for the red color of the pomegranate, are abundant in the arils. The peel contains a significant number of tannins like ellagic acid, pedunculagin, gallic acid, punicalin, and punicalagin; the flavonoids and phenolic chemicals present are catechins and anthocyanins. The most common anthocyanins are cyanidin-3-O-glucoside, cyanidin-3,5-di-O-glucoside, delphinidin-3-O-glucoside, pelargonidin-3-O-glucoside, and pelargonidin-3,5-di-O-gluco. Pomegranate arils also include phenolic acids, such as p-coumaric acid, chlorogenic acid, caffeic acid, ellagic acid, and gallic acid, as well as anthocyanins (8). Phenolic components, which are well recognized for their antioxidant property, are abundant in the peel. Proteins, organic acids, fibers, alkaloids, fatty acids, sugars (maltose, glucose, and fructose), potassium, phosphorus, sodium, calcium, and magnesium are present in the peel. The peel contains high amounts of isopelletierine, pseudopelletierine, pelletierine, and methylpelletierine, as well as alkaloids, succinic, citric, oxalic, and malic acids. It also contains indole amines, α-tocopherols, fatty acids, organic and amino acids, water (85%), pectins (1.4%), and sugar (10.6%) as well as triterpenoids but with a high number of phenols (0.2%–1%). The major groups of phenolic components of pomegranate contain hydrolyzed tannins like punicalagin, gallotanins, and ellagitannins. The fatty acids that are most prevalent in pomegranate seed oil are oleic, linoleic, and punicic acid. The majority of the seed is made up of 80% of punicic acid which is conjugated with 18 carbon fatty acids with triple bonds (9).

Thioredoxin-interacting protein (TXNIP) is an emerging regulator of cellular redox signaling closely intertwined with glucose and energy homeostasis. Over the past two decades, accumulating evidence has strongly established heightened TXNIP activity as a mediator of diabetes pathophysiology (10). TXNIP expression is drastically upregulated by elevated blood glucose levels and promotes insulin resistance through the inhibition of glucose uptake (11). Mechanistically, TXNIP binds with and inhibits the function of thioredoxin (TRX), an endogenous antioxidant factor, leading to a pro-oxidative shift in the intracellular environment (12). This redox imbalance then triggers downstream signaling cascades related to inflammation, gluconeogenesis, apoptosis, and other cell dysfunctions that collectively impair normal uptake and utilization of glucose, thereby worsening. Therefore, the ability of bioactive dietary plant compounds to target and inhibit TXNIP signaling has profound therapeutic implications in diabetes management. Uncovering these nutraceutical resources through innovative methods promises new treatment possibilities as emerging evidence continues to position TXNIP as the central molecular linchpin contributing to insulin resistance over progression to overt diabetes (13).

### Biological activities

Determining the antioxidant activity of natural components from flavors and substituting synthetic extracts to prevent the growth of some bacteria and antioxidants is therefore essential (14). Its inner network of membranes comprises almost 26%–30% of the fruit’s weight. Peel and juice compose approximately 92% of fruit antioxidant activity and are abundant sources of such chemicals. The water-soluble polyphenols, quality and safety of products, with preserving their anthocyanins and hydrolyzable tannins, are primarily responsible for this antioxidant capacity, which preservation techniques and to enhance the microbiological property (15).

The isomers of 2,3-(S)-hexahydroxydiphenoyl-4,6-(S,S)-gallagyl-D-glucose that make up the α and β forms of pomegranate punicalagin are polyphenolic hydrolyzed tannins. Pomegranate metabolizes into tiny polyphenol components in the intestinal tract during biological normal conditions because they are hydrophilic in nature (1).

Pomegranate peel showed antibacterial activities against *Escherichia coli*, *Listeria monocytogenes*, and *Staphylococcus aureus*. It has been demonstrated that a dose of 2 mg/disc of peel extract in association with ellagic acid has antimicrobial effects against *Streptococcus epidermidis*, *S. aureus*, and *Propionibacterium*. The peel worked as an antibacterial component when combined with lettuce and parsley before inoculation with *E. coli* and *S. aureus*. Similar research was conducted by Abdollahzadeh and partners on the impact of pomegranate methanol extract applied at 4, 8, and 12 mg/mL on the development of dental pathogens. Various tests performed on peel extracts showed effectiveness against *Streptococcus salivarius*, *Streptococcus mutans*, and *Lactobacillus acidophilus* and demonstrated resistance to *S. epidermidis* and *S. aureus*. The significant amount of ellagic acids and punicalagins present in extracts are responsible for inhibitory effects against *L. monocytogenes* through diffusion tests (16).

*L. monocytogenes* and *E. coli* showed antibacterial potential, but the peel also acts as a washing agent with hydrosol mint and mint essential oil. *L. monocytogenes* exhibited more potential than *E. coli* (17). Antibacterial assay was done through the agar well diffusion method and two types of bacteria were used for antimicrobial assay. *Pseudomonas aeruginosa* (ATCC 10145) and *E. coli* (ATCC 11775) were used as gram -ve bacteria while *S. aureus* (ATCC 6538) and *Bacillus subtilis* (ATCC 6051) were used as gram +ve bacteria (18). Pomegranate showed minimum inhibitory concentration (MIC) for antibacterial activity against *Bacillus cereus* and *S. aureus*. The peel improved the life span of chicken by 2–3 weeks by inhibiting the activity of *Pseudomonas* at a 0.1% dosage of the peel, but this therapy proved unsuccessful against *B. cereus* and *E. coli*. Studies proved that gram -ve and +ve bacteria showed antibacterial activities and the peel was used in the storage of beef. A strong antibacterial activity of films was observed against gram +ve bacteria when the peel was introduced to caseinate-based films (19).

### Health benefits

The peel includes hydrolyzable and condensed tannins, anthocyanins, hydroxycinnamic acid, and gallotannins. Punicalin, ellagitannins, and punicalagin are the phenolic compounds present in the peel that have anti-inflammatory, anti-mutagenic, anti-diabetic, antioxidant, wound healing, and antibacterial potential (15). The edible part and peel contain various compounds that have different health benefits. Gallagyl esters, anthocyanins, hydroxybenzoic acids, dihydroflavonol, gallotannins, hydroxycinnamic acids, and ellagitannins are the phenolic compounds in the peel that possess fruit antioxidant activity. Many studies have been published in the literature demonstrating the efficacy of ellagic acid as a treatment for numerous low to mild chronic diseases with extremely slow rates of development. Furthermore, it has proven to be effective for chemoprevention of cancer (20). Ellagic acid has been shown to reduce the production of triglycerides and white fat accumulation, which is a result of regular consumption of a high-fat diet, in addition to another of its ethno-pharmacological characteristics. The previous research found that oxidative damage, living cells, and reduction of sulfhydryl pool (non-protein) can be protected by the cytoprotective properties of the peel. The antioxidant activity of the peel is positively correlated with higher ellagic acid concentrations. The peel can also be used as a washing agent. NaOCl therapies and pomegranate extracts are used for cleaning fresh vegetables (21).

Cell damage is caused by the reaction of free radicals with carbohydrates, human DNA, lipids, and proteins because free radicals are highly unstable and volatile in nature (22). Free radicals react with macromolecules to promote carcinogenesis, nucleic acid oxidation, lipid peroxidation, and cardiovascular disorders. So, to prevent negative effects on human organs, tissues, and cells, antioxidants are used to inhibit the spontaneous free radical reaction (23). As an antioxidant-rich fruit, pomegranates can considerably and pleasantly impact people’s health. In addition to studies identifying phytochemicals present in pomegranate culture, the historical importance stimulates the research and evaluation of the anti-bacterial, anti-diabetic, and antioxidant properties of peel (24). Most food products need to be protected from phenolic antioxidants; during its shelf life, the peel is known to develop bacterial decomposition (25). With an increased consumer demand for safe herbal treatments with anti-bacterial, anti-atherosclerotic, anti-cancer, and wound healing capacities, researchers should thoroughly investigate the efficacy of the moderate antioxidant ability of pomegranate peel (15).

The bioactive compounds of the peel have shown antioxidant, antimicrobial, antidiabetic, and anti-inflammatory properties in various research, indicating their therapeutic effects in different diseases such as ethanol-acetone ulceration, gastro-mucosal injuries, diabetic oxidative damage, and leukemia chemoprevention. Precipitation of membrane proteins that results in microbial cell death is the mechanism through which peel phenolics exhibit their antibacterial effect. Peel anti-mutagenic and antibacterial characteristics are part of its ethno-medicinal profile that makes its traditional value. Furthermore, phytochemicals present in the peel are effective without adding extracts from any other fruit components (26). The pomegranate fruit and its derivatives may contain medicinal value with significant phytochemicals and secondary metabolites, according to previous research. Oxidative food stability can be maintained by using peel extract. *Litopenaeus vannamei* is prevented using peel extract in methanol as it works like a natural preservative. Two doses of peel extracts of 10 or 20 g significantly inhibit the growth of *Enterobacteriaceae*, psychrophilic, mesophilic, and lactic acid bacteria (LAB) in white shrimps. Pomegranate peel stabilizes shrimp quality and is used to decrease thiobarbituric, trimethylamine, and total volatile basic nitrogen. It is used to preserve Kalari cheese in India. The peel inhibits the production of coliform during preservation because fungus, yeast, and psychrophilic quantities are inhibited by peel extracts. The aims and objectives of the research include the preparation of extracts in different solvents such as methanol, chloroform butanol, and *n*-hexane. Phytochemical identification was done and gas chromatography-mass spectrometry (GC*-*MS) analysis of extracts was performed. The biological activities of the peel were determined. *In silico* studies were performed to design drugs against diabetes (8).

## Materials and methods

Pomegranate fruits were obtained from local markets and peels were separated, cut, dried at room temperature, and powdered using a commercial grinder. For each prepared extract, 50 g of pomegranate peel powder was soaked in 500 mL of solvent (methanol, butanol, *n*-hexane, or chloroform) for 10 days at room temperature. The extracts were then filtered, and the filtrates were dried first using a rotary evaporator at 45°C followed by a drying oven to remove any remaining solvent. This yielded dried methanolic, butanolic, hexanic, and chloroform pomegranate peel extracts for subsequent phytochemical analysis and bioactivity testing experiments (see Table 2).

**Table 2:** Phytochemical screening of *P. granatum* peel.

Sr.	Tests	Plant extracts			
		Methanol	Butanol	*n*-hexane	Chloroform
1.	Phenols	+	−	+	−
2.	Flavonoids	+	+	−	+
3.	Glycosides	+	+	−	+
4.	Tannins	+	+	+	−
5.	Terpenoids	+	+	−	+
6.	Steroids	+	−	+	−
7.	Saponins	−	+	+	−

### Phytochemical screening of extracts

#### Test for phenols

A pinch of FeCl_3_ and 3 mL ethanol were added to 2 mL of extract. Phenols were confirmed by the appearance of greenish-yellow coloration (27).

#### Test for flavonoids

One milliliter of each extract was added into the test tube then 1 mL of 10% NaOH was added. The presence of flavonoids was confirmed by the appearance of yellow to orange color.

#### Test for tannins

One milliliter of each extract was added to different test tubes then 1 mL of FeCl_3_ was added. The presence of tannins was confirmed by the appearance of blue to green color.

#### Test for saponins

Distilled water (20 mL) was added to 2 mL of sample in a test tube and shaken for 15 min.

Layers of 1 cm foam appeared and saponins were indicated.

#### Test for terpenoids

In the test tubes, 2 mL of each extract and 3 mL of conc were added. H_2_SO_4_ was added along the wall of the test tube. Then, 2 mL of chloroform was added and there was the formation of single reddish-brown layers appeared and the presence of terpenoids was confirmed.

#### Test for glycosides

**Figure 2:**
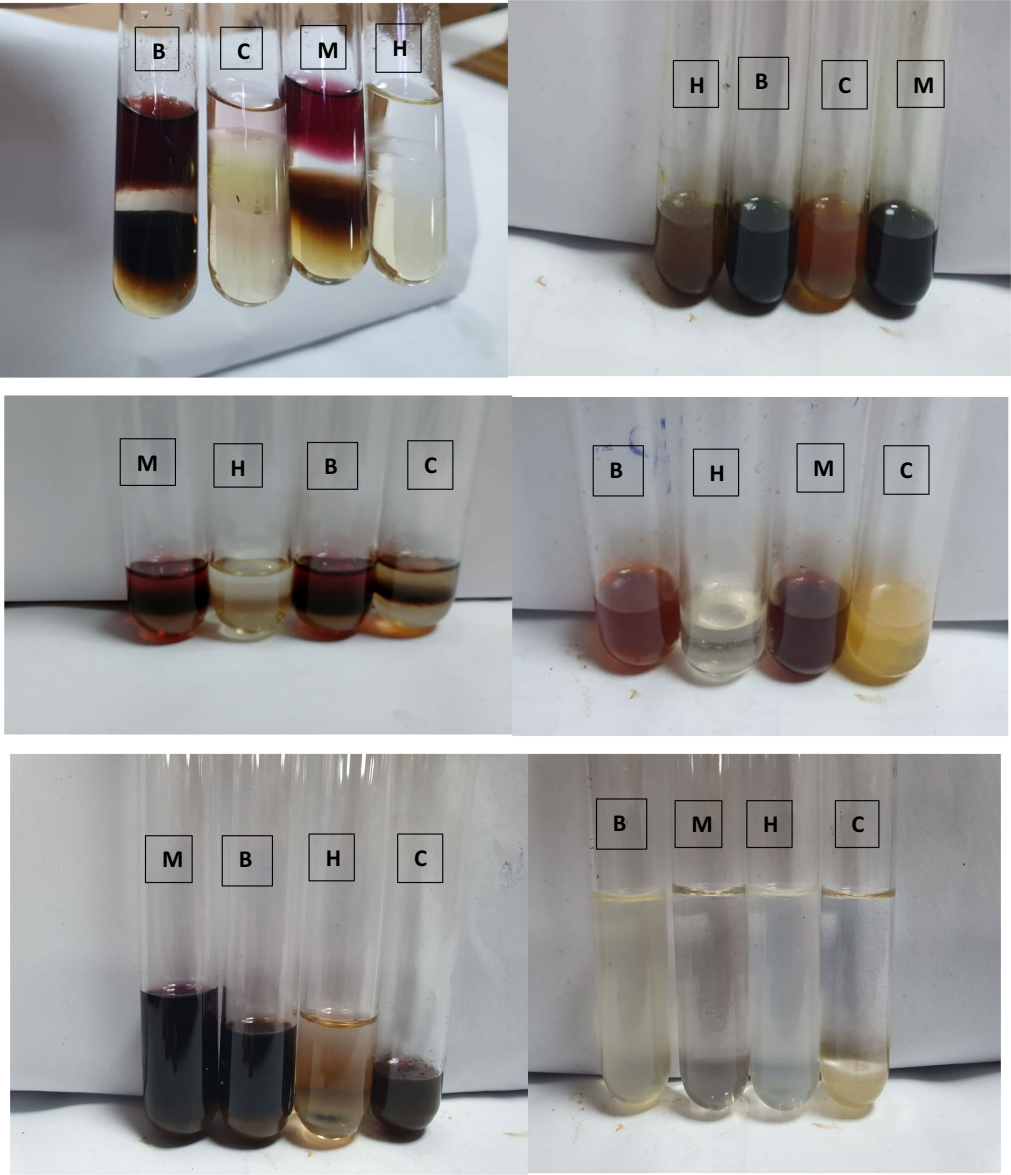
Phytochemical screening of *P. granatum* peel extract [M (methanol), B (butanol), H (*n*-hexane), and C (chloroform)].

In each test tube, 0.5 mg of each extract was added to 1 mL of distilled water and 1 mL of aqueous NaOH was added (Figure 2). Glycosides were confirmed by the formation of a yellow color (28).

### Biological activities of *Punica granatum*

#### DPPH radical scavenging activity

DPPH radical scavenging assay was carried out to evaluate antioxidant potential. A 0.5 mg/mL DPPH solution was prepared in ethanol. Different concentrations of the pomegranate peel extract (0.5, 0.25, 0.125, 0.0625, and 0.03125 mg/mL) were prepared in DMSO. In an ELISA plate protected from light, 100 μL DPPH solution was mixed with an equal volume of the extract solutions and incubated at 37°C for 35 min. DMSO solution served as the control. Absorbance was measured at 630 nm. Standard ascorbic acid solutions were processed in parallel for comparison along with a negative control lacking extract. All experimental and standard conditions were performed in triplicates. Decreased absorbance indicated free radical scavenging through hydrogen donation and the antioxidant capacity of extracts. The IC50 values denoted effective concentrations of extract displaying 50% radical inhibition (28).

The formula used to calculate the radical scavenging percentage is:


$$\begin{array}{l} \mathrm{Percentage radical scavenging activity}\\ =\left[\left(\mathrm{Absorbance of control}-\mathrm{Absorbance of sample}/\mathrm{Absorbance of control}\right)\times 100\right] \end{array}$$

#### Anti-inflammatory activity

The anti-inflammatory activity of *P. granatum* extract and its fractions was assessed using the method described with modifications. For the preparation of bovine serum albumin protein solution, 200 mg BSA was dissolved in 20 mL distilled water. Twenty Eppendorf were taken in which 100 μL of each concentration solution of extract (0.5, 0.25, 0.125, 0.0625, and 0.03125 mg/mL) was taken; then in all Eppendorf, 100 μL BSA solution was added. After this, they were heated in a water bath for some time at 70°C. A measurement of 100 μL of mixture solution was then added to an ELISA plate and its absorbance was measured at a wavelength of 630 nm. A dicloran concentration of 0.1 mg/mL was taken as the positive control and distilled water as the negative control. Triplicate values were taken. The formula used to calculate the inhibition percentage (28) is:


$$\begin{array}{l} \mathrm{Percentage anti}-\mathrm{inflammatory activity}\\ =\left[\left(1-\mathrm{Absorbance of sample}/\mathrm{Absorbance of negative control}\right)\times 100\right] \end{array}$$

#### Anti-diabetic activity

The anti-diabetic activity was assessed by α-glucosidase assay. Phosphate buffer with 0.07 M molarity and 6.8 pH was prepared with *p*-NPG substrate, α-glucosidase enzyme (2.0 U m/L), and α-glucosidase (2.5 U m/L). In the ELISA plate, 70 μL of buffer was added in the wells and then incubated for 5 min. After that, 10 μL of extracts and 10 μL of enzymes were added to each well. Substrate of 10 μL was added and re-incubated for 30 min. After that, 80 μL of Na_2_CO_3_ was added as a negative control to stop the reaction. Acarbose was used as a positive control. Then anti-diabetic potential against α-glucosidase was measured using ELISA at 405 nm (29). Results were obtained using the following formula:


$$\begin{array}{l} \mathrm{Inhibition}\%=\\ \left[\left(1-\mathrm{Absorbance of sample}/\mathrm{Absorbance of control}\right)\times 100\right] \end{array}$$

#### Antibacterial activity

Antibacterial assay of *P. granatum* samples was performed by the disc diffusion method. Both gram -ve and gram +ve bacterial strains were used to find out the antibacterial activity. *Staphylococcus viridans* strain was used as gram +ve and *E. coli* strain was used as gram -ve.

##### Preparation of culture

We used nutrient broth for the culture preparation. One gram of NaCl, 1 g of pet one, and 0.5 g of yeast were dissolved in 100 mL of distilled water. After proper mixing, a yellow color was formed and the nutrient broth was autoclaved. Then, the nutrient broth was equally poured into three conical flasks after cooling (25°C). One colony of bacteria was picked using a sterile loop and transferred into falcon tubes; all experiments were performed in laminar flow. The broth was placed in a shaking incubator (37°C) for 24 h. After 24 h, faint turbidity appeared and the culture was prepared.

##### Preparation of plates

Preparation of plates was done using 2 g of NaCl, 2 g of peptone, 1 g of yeast, and 4 g of agar dissolved in 200 mL of distilled water. Then, the material and plates were autoclaved to avoid contamination. After that, the experiment was performed in laminar flow. Plates were poured and kept for a while for solidification.

##### Spreading

The culture was picked up with cotton buds and then spread on plates.

##### Disc diffusion method

The antibacterial activity of four extracts of *P. granatum* was investigated by the disc diffusion method. Bacterial strains were used for this purpose. Strains were picked up and spread on agar plates. Whatman filter paper with 11 μm pore size was dipped in the media plates with 200 mg/mL of extract concentration. Then, the plates were kept in an incubator for 24 h at 37°C by covering properly with paraffin. DMSO solution (10%) was used as a negative control and ciprofloxacin was used as a positive control. Then, after 24 h, plates were checked and the zone was measured in mm (30).

### Drug designing against diabetes

#### Protein structure retrieval from Protein Data Bank

A Protein Data Bank is a database that contains a large number of protein sequences, structures, and functions. The 3D structure of the required protein, i.e., thioredoxin interaction protein, was retrieved from PDB. The selected protein PDB ID is 1-TRW (31).

#### Ligand structure retrieval from PubChem

The National Center of Biotechnology Information (NCBI) has established a PubChem server for ligand retrieval. Some other servers that can also be used for ligand retrieval are ZINC and ChEMBL. Ligands were downloaded as a 3D SDF file and different ligands were taken from PubChem (32).

#### Preparation of ligand and protein by Discovery Studio Visualizer

Discovery Studio Visualizer was used for the preparation of protein and ligands. For the ligand and protein purification, water molecules were removed and the file was saved from the SDF to the PDB file. So, purified protein and ligand were obtained (33).

#### Molecular docking of ligand and protein

AutoDock Vina is the most used software for checking the interaction between proteins and ligands with each other. It provides a complex structure with complete interaction of ligand to protein. First, the purified structure of protein is opened in AutoDock Vina then the grid is set out with dimensions and a torsion tree. Then, the command is run and energies of different values are taken (34) (see Figure 2).

## Results

### Qualitative analysis of *Punica granatum*

Phytochemical analysis showed that phenols were present in methanol and *n*-hexane extracts while other extracts showed negative results. Flavonoids were present in all extracts except in *n*-hexane. All extracts contained glycosides except *n*-hexane. Tannins were present in methanol, butanol, and *n*-hexane but not in chloroform extract. Terpenoids were present in all extracts but not in *n*-hexane extracts. Steroids only showed in methanol and *n*-hexane extracts. Saponins were present in butanol and *n*-hexane extracts but were not present in methanol and chloroform extracts.

### Quantitative analysis of methanol and *n*-hexane extract through gas chromatography-mass spectrometry

**Figure 3:**
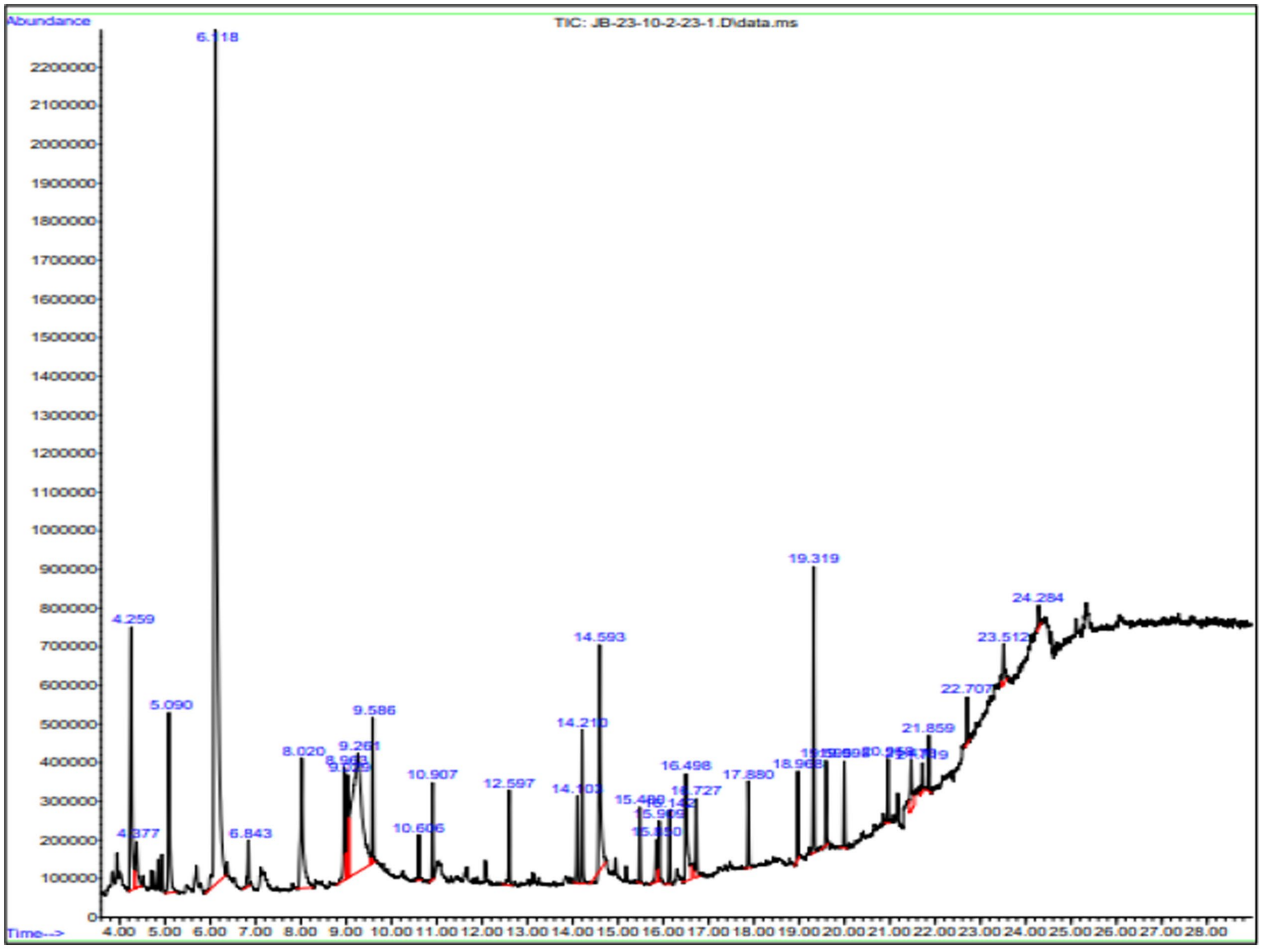
GC*-*MS chromatogram of methanol extract of *P. granatum*. Methanolic extracts have 43 compounds, and retention time, peaks, and peak area were determined by GC*-*MS.

Gas chromatography connected with mass spectroscopy was used to determine the methanolic extract of *P. granatum* peel. It shows many compounds that have antioxidant, anti-diabetic, and anti-inflammatory properties. The highest-placed compounds were examined by GC*-*MS. GC*-*MS spectrum showed the 43 compounds with their chemical structure, retention time, and area. The identification of compounds is shown in Figure 3.

The compounds that have been identified in methanol extract, their names, formulas, molecular weight, and retention time are presented in Table 3.

**Table 3:** Compounds identified in methanol extract by GC*-*MS.

Sr.	Compound name	Mol. formula	Mol. weight (g/mol)	Retention time (mint)
1.	Thymine	C_5_H_6_N_2_O_2_	126.11	4.259
2.	3,5-octadien-2-one	C_8_H_12_O	124.18	4.377
3.	4H-Pyran-4-one, 2,3-dihydro-3,5-dihydroxy-6-methyl	C_6_H_8_O_4_	144.12	5.090
4.	1H-pyrrole-2,5-dione	C_4_H_3_NO_2_	97.07	6.118
6.	*n*-propylcyclopropanemethylamine	C_7_H_15_N	113.20	8.020
7.	Trisiloxane, 1,1,1,5,5,5-hexamethyl-3,3-bis[(trimethylsilyl)oxy]-	C_12_H_36_O_4_Si_5_	384.84	8.963
8.	Beta-D-lyxofuranoside, 5-O-(beta-D-lyxofuranosyl)-decyl	C_20_H_38_O_9_	422.5	9.029
10.	Butylated hydroxytoluene	C_15_H_24_O	220.35	9.586
11.	Diethyl phthalate	C_12_H_14_O_4_	222.24	10.606
12.	Benzeneethanamine, N-[(pentafluorophenyl)methylene]-beta.,3,4-tris[(trimethylsilyl)oxy]-	C_24_H_34_F_5_NO_3_Si_3_	563.8	10.907
13.	(S)-(-)-1,1′-Bi-2-naphthol, O,O′-bis(trimethylsilyl)	C_26_H_30_O_2_Si_2_	430.7	12.597
14.	2-(2′,4′,4′,6′,6′,8′,8′-Heptamethyltetrasiloxan-2′-yloxy)-2,4,4,6,6,8,8,10,10-nonamethylcyclopentasiloxane	C_16_H_48_O_10_Si_9_	653.3	14.103
15.	Hexadecanoic acid, methyl ester	C_17_H_34_O_2_	270.5	14.210
16.	*n*-hexadecanoic acid	C_16_H_32_O_2_	256.42	14.593
17.	Hexasiloxane, tetradecamethyl	C_14_H_42_O_5_Si_6_	458.99	15.480
18.	9,12-octadecadienoic acid (Z,Z)-, methyl ester	C_19_H_34_O_2_	294.5	15.850
19.	9-octadecenoic acid (Z)-, methyl ester	C_19_H_36_O_2_	296.5	15.909
20.	Heptadecanoic acid, 16-methyl-, methyl ester	C_19_H_38_O_2_	298.5	16.142
21.	Octadecanoic acid	C_18_H_36_O_2_	284.5	16.498
22.	Hexasiloxane, tetradecamethyl	C_14_H_42_O_5_Si_6_	458.99	16.727
23.	Trisiloxane, 1,1,1,5,5,5-hexamethyl-3,3-bis[(trimethylsilyl)oxy]	C_12_H_36_O_4_Si_5_	384.84	18.968
24.	Oxalic acid, 2-ethylhexyl tetradecyl ester	C_24_H_46_O_4_	398.6	19.319
25.	Bis(2-ethylhexyl) phthalate	C_24_H_38_O_4_	390.6	19.595
26.	Octasiloxane, 1,1,3,3,5,5,7,7,9,9,11,11,13,13,15,15-hexadecamethyl	C_16_H_48_O_7_Si_8_	577.2	20.958
27.	Tetrasiloxane, decamethyl	C_10_H_30_O_3_Si_4_	310.68	22.707
28.	Methyltris(trimethylsiloxy)silane	C_10_H_30_O_3_Si_4_	310.68	23.512

#### GC*-*MS analysis of *n*-hexane

**Figure 4:**
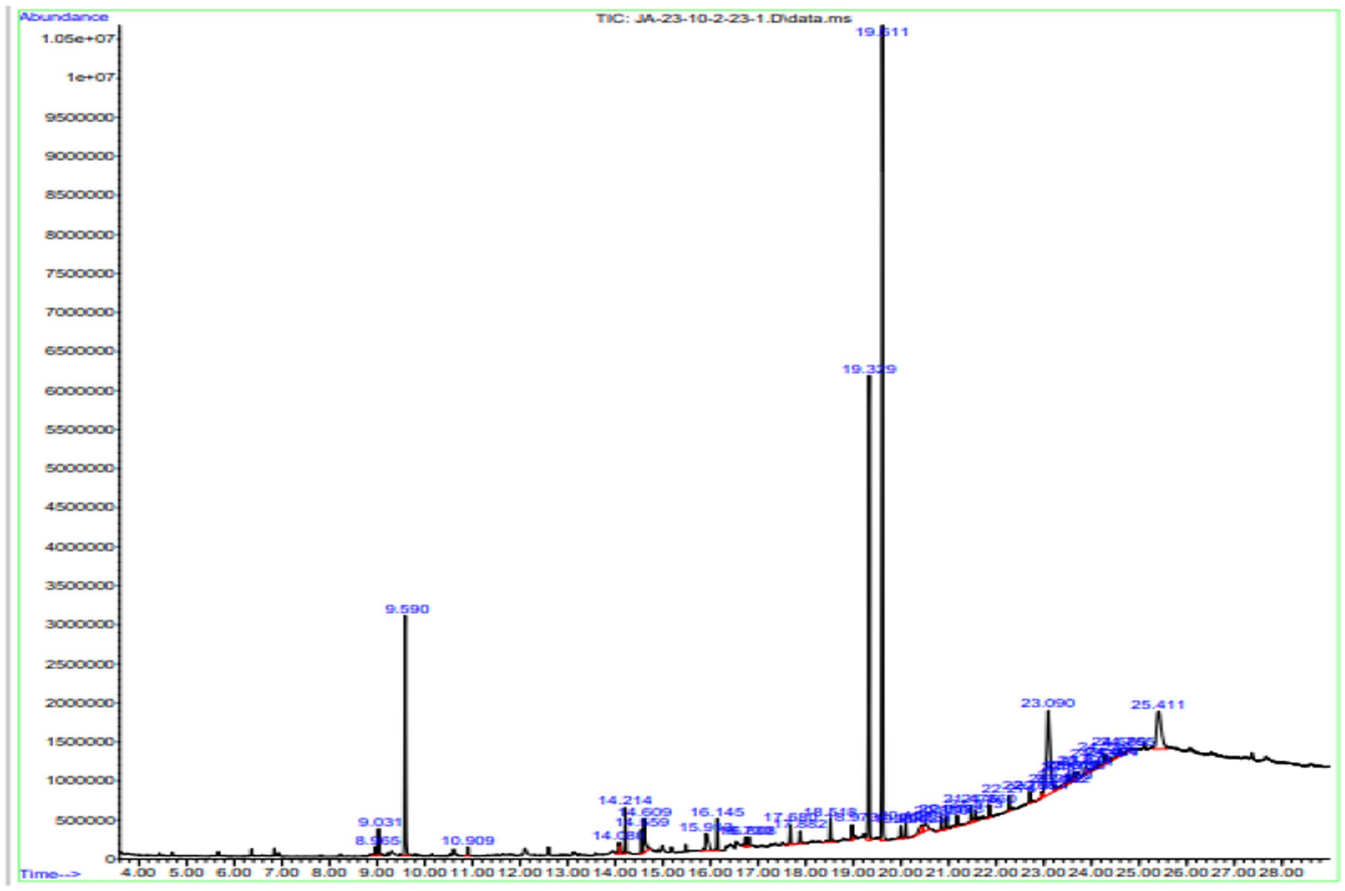
GC*-*MS chromatogram of *n*-hexane extract of *P. granatum*. *n*-hexane extract has 42 compounds, and retention time, peaks, and peak area were determined by the GC*-*MS.

Gas chromatography connected with mass spectroscopy was used to determine the methanolic extract of *P. granatum* peel. It was used to determine many compounds that have antioxidant, anti-diabetic, and anti-inflammatory properties. The highest-placed compounds were examined by GC*-*MS. GC*-*MS spectrum showed the 42 compounds with their chemical structure, retention time, and area. Octasiloxane, 1,1,3,3,5,5,7,7,9,9,11,11,13,13,15,15-hexadecamethyl, butylated hydroxytoluene, *n*-hexadecanoic acid, 9,12-octadecadienoic acid (Z,Z), Carbonic acid, dodecyl 2-ethylhexyl ester, *cis*-9-hexadecenoic acid, tetradecanoic acid, and 2-Heptenoic acid were obtained with highest peaks. The identified compounds are presented in Figure 4.

The compounds identified in *n*-hexane extract, their compound names, formulas, molecular weight, and retention time are shown in Table 4.

**Table 4:** Compounds identified in *n*-hexane extract by GC*-*MS.

Sr.	Compound name	Mol. formula	Mol. Weight (g/mol)	Retention time (mint)
1.	Octasiloxane, 1,1,3,3,5,5,7,7,9,9,11,11,13,13,15,15-hexadecamethyl	C_16_H_48_O_7_Si_8_	577.2	8.965
2.	2,6-Di-tert-butyl-4-hydroxy-4-methylcyclohexa-2,5-dien-1-one	C_15_H_24_O_2_	236.35	9.031
3.	Butylated hydroxytoluene	C_15_H_24_O	220.35	9.590
4.	Benzeneethanamine, N-[(pentafluorophenyl)methylene]-.beta.,3,4-tris[(trimethylsilyl)oxy]	C_24_H_34_F_5_NO_3_Si_3_	563.8	10.909
5.	7,9-Di-tert-butyl-1-oxaspiro(4,5)deca-6,9-diene-2,8-dione	C_17_H_24_O_3_	276.4	14.080
6.	Hexadecanoic acid, methyl ester	C_17_H_34_O_2_	270.5	14.214
7.	1,4-Dibutyl benzene-1,4-dicarboxylate	C_16_H_22_O_4_	278.34	14.559
8.	*n*-hexadecanoic acid	C_16_H_32_O_2_	256.42	14.609
9.	Cyclohexadecane	C_16_H_32_	224.42	15.913
11.	1,1,1,5,7,7,7-heptamethyl-3,3-bis(trimethylsiloxy)tetrasiloxane	C_13_H_39_O_5_Si_6_	443.96	16.730
12.	Decane, 3,8-dimethyl	C_9_H_14_N_2_O_3_	198.22	16.808
14.	Octasiloxane, 1,1,3,3,5,5,7,7,9,9,11,11,13,13,15,15-hexadecamethyl	C_16_H_48_O_7_Si_8_	577.2	17.882
17.	Carbonic acid, dodecyl 2-ethylhexyl ester	C_21_H_42_O_3_	342.6	19.329
18.	Bis(2-ethylhexyl) phthalate	C_24_H_38_O_4_	390.6	19.611
19.	Methyltris(trimethylsiloxy)silane	C_10_H_30_O_3_Si_4_	310.68	19.996
20.	Tetradecane, 2,6,10-trimethyl	C_17_H_36_	240.5	20.101
21.	Arsenous acid, tris(trimethylsilyl) ester	C_9_H_27_AsO_3_Si_3_	342.49	20.422
22.	Arsenous acid, tris(trimethylsilyl) ester	C_9_H_27_AsO_3_Si_3_	342.49	20.531
23.	Methyltris(trimethylsiloxy)silane	C_10_H_30_O_3_Si_4_	310.68	20.850
24.	4-(4-Hydroxyphenyl)-4-methyl-2-pentanone	C_15_H_24_O_2_Si	264.43	20.958
28.	Tetrasiloxane, decamethyl	C_10_H_30_O_3_Si_4_	310.68	21.860
29.	Cyclotrisiloxane, hexamethyl	C_6_H_18_O_3_Si_3_	222.46	22.274
31.	1,2-bis(trimethylsilyl)benzene	C_12_H_22_Si_2_	222.47	22.954
32.	Methyltris(trimethylsiloxy)silane	C_10_H_30_O_3_Si_4_	310.68	23.090
33.	Arsenous acid, tris(trimethylsilyl) ester	C_9_H_27_AsO_3_Si_3_	342.49	23.252
34.	Arsenous acid, tris(trimethylsilyl) ester	C_9_H_27_AsO_3_Si_3_	342.49	23.402
36.	4-(7-methyloctyl)phenol	C_15_H_24_O	220.35	23.512
39.	2,4,6-cycloheptatrien-1-one, 3,5-bis-trimethylsilyl	C_13_H_22_OSi_2_	250.48	23.874
40.	Tetrasiloxane, decamethyl	C_10_H_30_O_3_Si_4_	310.68	23.911
41.	4-(4-hydroxy-2,5-dimethylbenzyl)morpholine, TMS			24.134
42.	Arsenous acid, tris(trimethylsilyl) ester	C_9_H_27_AsO_3_Si_3_	342.49	24.286

### Biological activities of *Punica granatum*

#### Antioxidant activity

**Figure 5:**
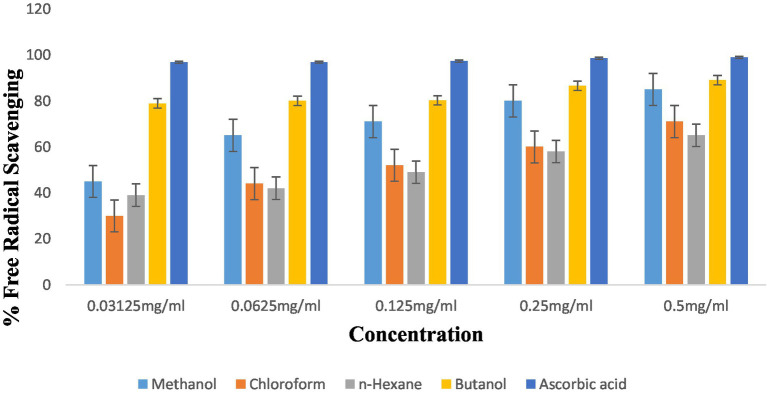
Antioxidant activity of *P. granatum* extracts.

The antioxidant potential of *P. granatum* and its fractions was determined using the DPPH radical scavenging assay. Radical scavenging activity was evaluated at different concentrations of fractions. Results were analyzed statistically. Methanol, chloroform, *n*-hexane, and butanol showed antioxidant potential at 0.5 mg/mL concentrations of 85 ± 0.096, 71 ± 0.029, 65 ± 0.050, and 89 ± 0.019, respectively. When concentration decreased, then antioxidant potential also decreased to 80 ± 0.029, 60 ± 0.025, 58 ± 0.049, and 86.6 ± 0.023 for methanol, chloroform, *n*-hexane, and butanol concentration, respectively. Butanol and methanol fractions showed the highest antioxidant activity. The order of antioxidant activity results was butanol > methanol > chloroform > *n*-hexane. Results are shown in Figure 5. Ascorbic acid was taken as standard which showed radical scavenging percentage at different concentrations, i.e., 0.5, 0.25, 0.125, 0.0625, and 0.03125 mg/mL as 98.9 ± 0.007, 98.5 ± 0.005, 97.3 ± 0.012, 96.6 ± 0.015, and 96 ± 0.014, respectively.

The antioxidant activity of peel has been shown by different extracts. The highest activity was shown by butanol and methanol extract which are 89 ± 0.019 and 85 ± 0.096, respectively, as shown in Figure 5.

#### Anti-inflammatory activity

**Figure 6:**
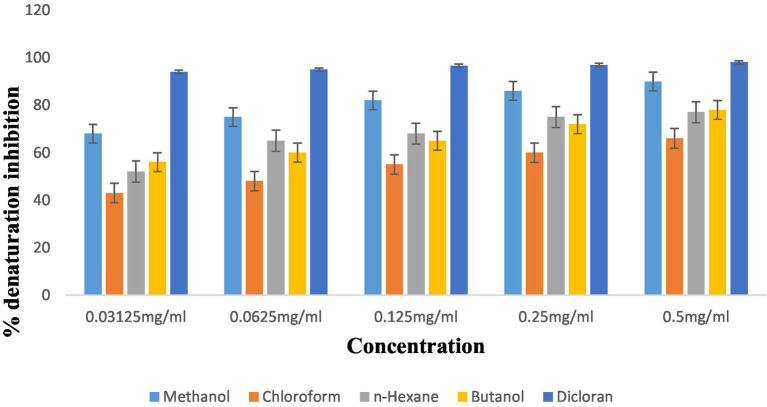
% age inhibition of *P. grantum* extracts.

Results showed that fractions at concentrations of 0.5 mg/mL extracts such as methanol, chloroform, *n*-hexane, and butanol showed anti-inflammatory activity at 90 ± 0.034, 66 ± 0.017, 77 ± 0.060, and 78 ± 0.017, respectively. When concentration decreased, inhibition values also decreased. At 0.25 mg/mL, inhibition decreased at 86 ± 0.016, 60 ± 0.020, 75 ± 0.045, and 72 ± 0.009, respectively according to fractions. Dicloran was taken as standard which showed its maximum inhibition at 98 ± 0.005, 97.8 ± 0.015, 96.6 ± 0.018, 95 ± 0.019, and 94 ± 0.012 at different concentrations of 0.5, 0.25, 0.125, 0.625, and 0.03125 mg/mL, so dicloran exhibited inhibition percentages of 98%, 97.8%, 96.6%, 95%, and 94%. Results are presented in Figure 6.

The anti-inflammatory activity of the plant has been shown in different extracts at different concentrations. The highest activity was shown by methanol and butanol at 90 ± 0.034 and 78 ± 0.017, respectively, and results are shown in Figure 6.

#### Antibacterial activity

**Figure 7:**
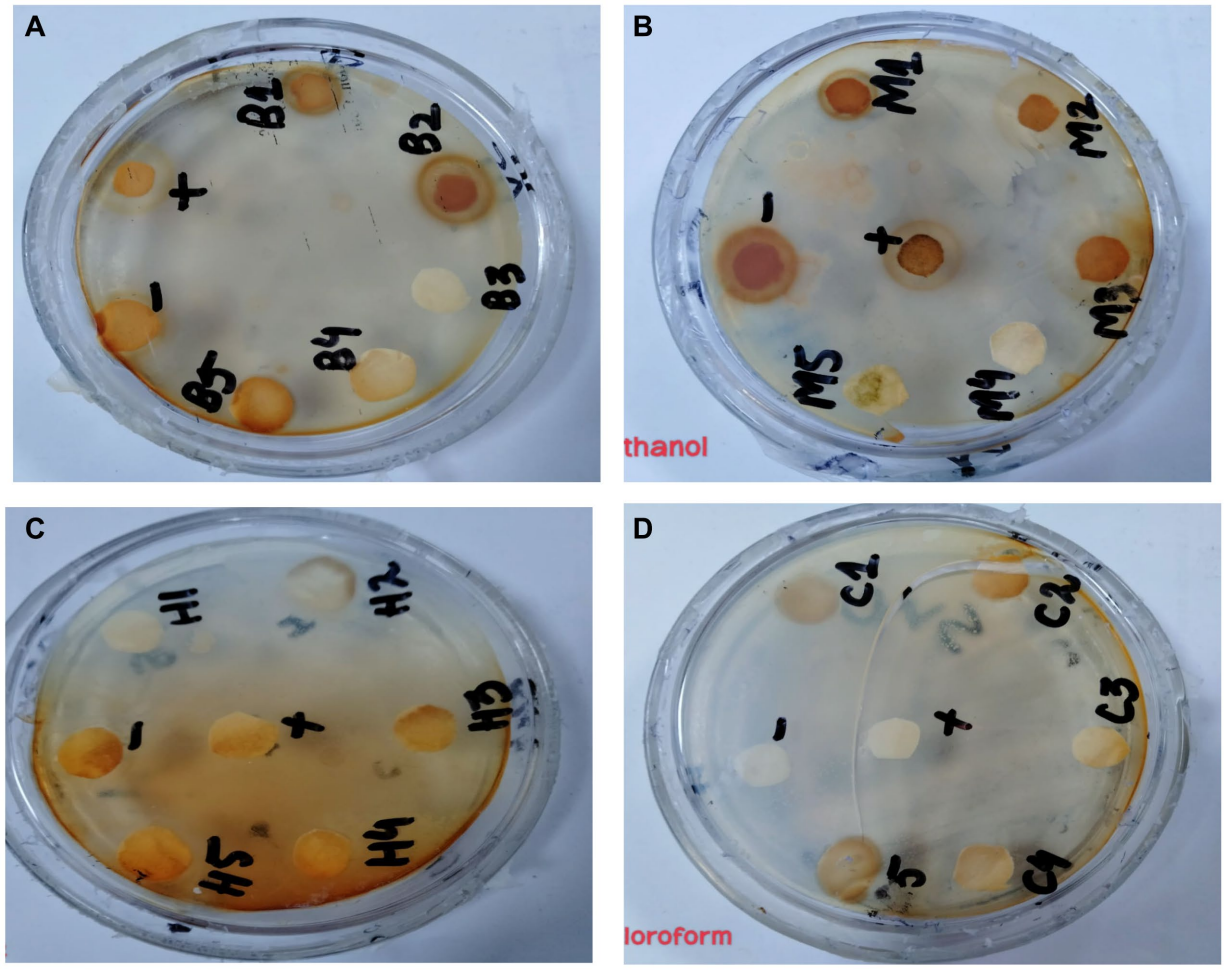
Antibacterial effect of extracts against resistant bacteria *S. viridans* **(A)** butanol, **(B)** methanol, **(C)** *n*-hexane, and **(D)** chloroform.

**Figure 8:**
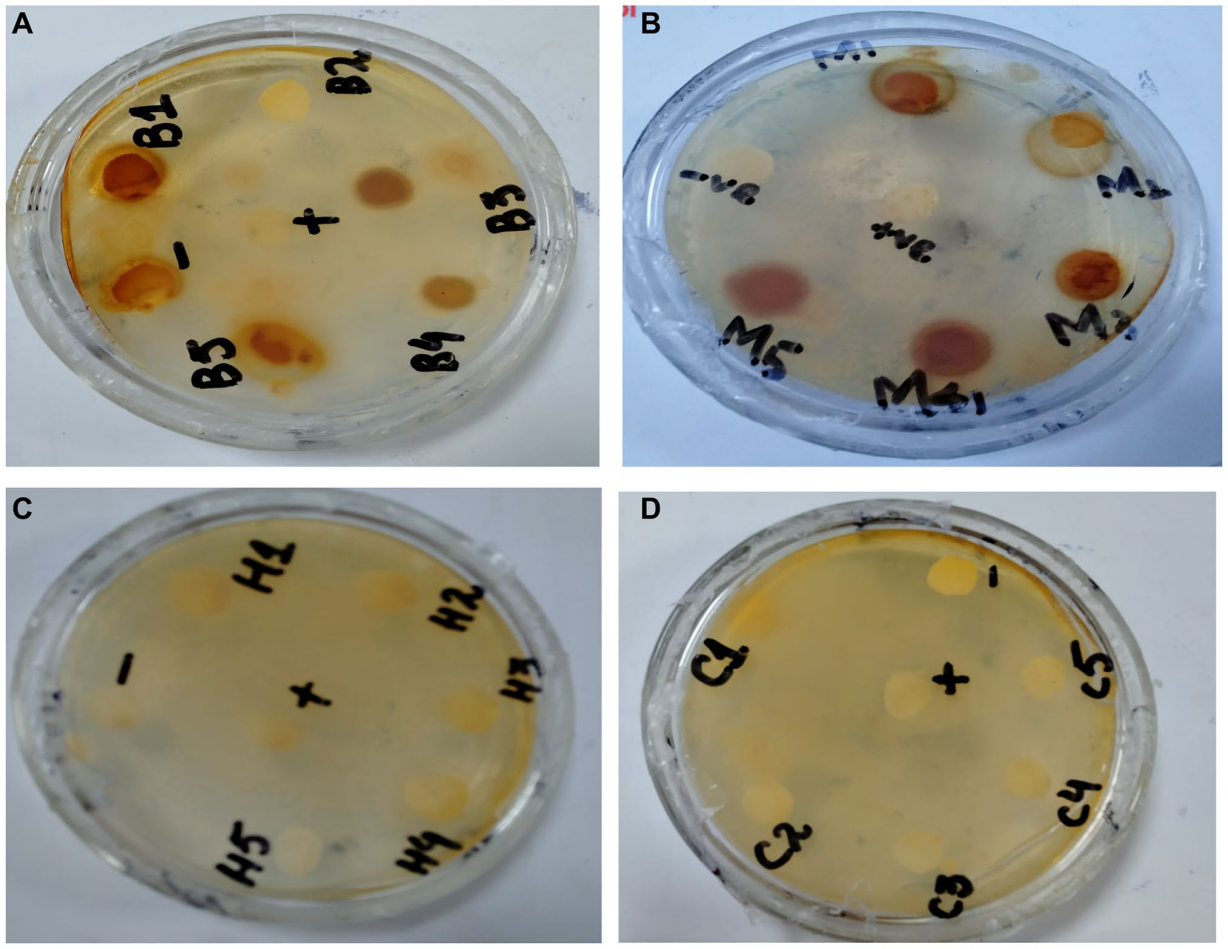
Non-resistant bacteria *E. coli* **(A)** methanol, **(B)** butanol, **(C)** *n*-hexane, and **(D)** chloroform.

In the antibacterial assay, *S. viridans* was used as gram +ve and *E. coli* as gram -ve bacterial strains. Extracts of methanol and butanol showed resistance against gram +ve bacteria. Peel extract of methanol and butanol showed antibacterial activity against *S. viridans*. The zone of inhibition has been measured in mm for different extracts of *P. granatum* peel, as shown in Table 5. The zones shown in Figure 7 are for *S. viridans*. The non-resistant bacteria were *E. coli* whose results are shown in Figure 8.

**Table 5:** Antibiotics resistance against various resistant and non-resistant bacteria.

Sr.	Bacterial strains	Extracts	Concentration (mg/mL)	Mean ± S.D.
1.	*Staphylococcus viridans*	Methanol	0.5	15 ± 0.34
			0.25	7 ± 0.15
			0.125	12 ± 0.26
			0.0625	10 ± 0.22
			0.03125	13 ± 0.29
		Butanol	0.5	16 ± 0.39
			0.25	10 ± 0.22
			0.125	12 ± 0.26
			0.0625	8 ± 0.16
			0.03125	6 ± 0.14
		*n*-hexane	0.5	0
			0.25	2 ± 0.06
			0.125	3 ± 0.09
			0.0625	8 ± 0.16
			0.03125	0
		Chloroform	0.5	10 ± 0.22
			0.25	0
			0.125	0
			0.0625	0
			0.03125	12 ± 0.26
		Ampicillin	+ve control	16 ± 0.39
		10% DMSO	−ve control	0
2.	*E. coli*	Methanol	0.5	8 ± 0.16
			0.25	10 ± 0.22
			0.125	6 ± 0.14
			0.0625	7 ± 0.15
			0.03125	3 ± 0.09
		Butanol	0.5	10 ± 0.22
			0.25	6 ± 0.14
			0.125	9 ± 0.20
			0.0625	8 ± 0.16
			0.03125	3 ± 0.09
		*n*-hexane	0.5	0
			0.25	0
			0.125	0
			0.0625	0
			0.03125	0
		Chloroform	0.5	0
			0.25	0
			0.125	0
			0.0625	0
			0.03125	0
		Ampicillin	+ve control	0
		10% DMSO	−ve control	0

#### Anti-diabetic activity

**Figure 9:**
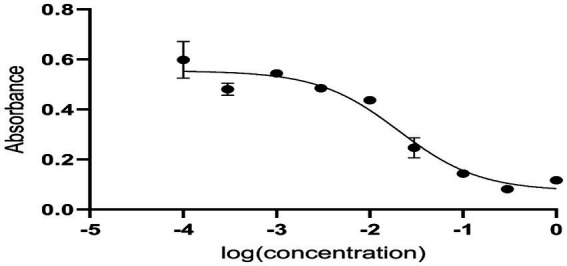
Determination of IC_50_ of methanol extract.

**Figure 10:**
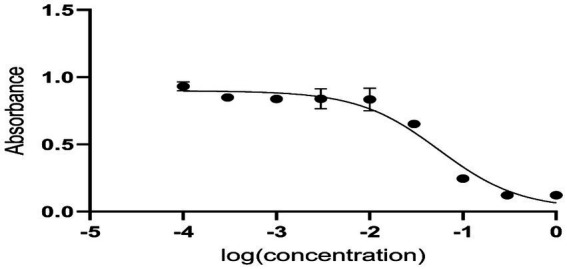
Determination of IC_50_ of butanol extract.

**Figure 11:**
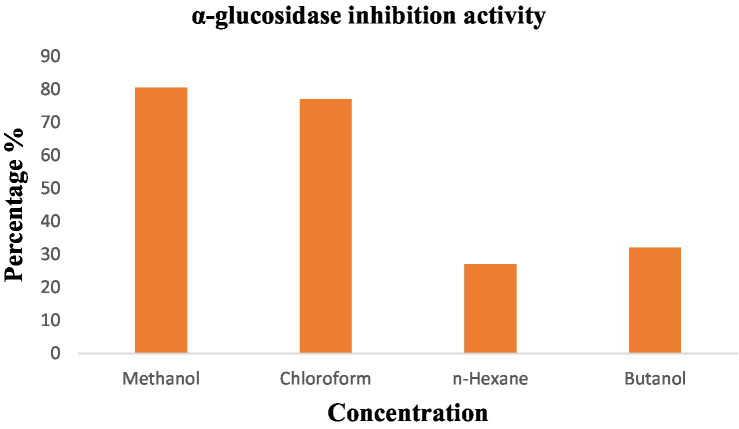
Graphical representation of anti-diabetic results of *P. granatum.*

Anti-diabetic activity was done using ELISA. The highest activity was observed in methanol, and butanol showed the lowest activity. The greatest activity was exhibited by methanol and butanol extracts. Determination of IC_50_ of methanol and butanol extracts is shown in Figures 9–11.

The percentage concentration of extracts is shown in Table 6. The highest percentage of methanol extract is 80.5%.

**Table 6:** Energies of TXNIP protein with 3,5-octadien-2-one.

Mode	Binding affinity (kcal/mol)	Distance from the best mode	
		(rmsd 1.b.)	(rmsd u.b.)
**Binding affinity of 3,5-octadien-2-one with TXNIP**			
1	−3.9	0.000	0.000
2	−3.8	18.621	19.601
3	−3.8	2.839	3.220
4	−3.7	3.181	5.382
5	−3.7	23.481	24.945
6	−3.6	14.582	15.811
7	−3.5	3.641	5.815
8	−3.4	4.053	4.850
**Binding affinity of 1H-pyrrole -2,5-dione with TXNIP**			
1	−3.9	0.000	0.000
2	−3.6	15.542	15.954
3	−3.6	15.541	16.167
4	−3.6	16.911	17.284
5	−3.6	1.864	2.169
6	−3.5	15.321	15.593
7	−3.4	15.345	15.890
8	−3.4	15.515	16.114
9	−3.4	18.617	18.985
**Binding affinity of 1H-pyrrole -2,5-dione beta-D-lyxofuranoside, 5-O-(beta-D-lyxofuranosyl)-decyl with TXNIP**			
1	−4.4	0.000	0.000
2	−4.3	17.474	20.481
3	−4.2	16.557	18.937
4	−4.2	1.458	4.922
5	−4.0	1.590	3.029
6	−3.9	19.676	23.532
7	−3.9	1.410	4.955
8	−3.7	24.814	28.370
9	−3.6	1.257	2.171
**Binding affinity of diethyl phthalate with TXNIP**			
1	−4.2	0.000	0.000
2	−4.2	26.476	30.028
3	−4.2	1.757	3.085
4	−4.1	4.055	7.863
5	−4.1	1.584	2.253
6	−4.0	1.471	2.514
7	−3.9	11.158	13.499
8	−3.8	22.068	23.333
9	−3.7	1.557	3.513
**Binding affinity of 9-octadecenoic acid (Z)-, methyl ester**			
1	−4.5	0.000	0.000
2	−4.4	20.879	23.520
3	−4.4	20.350	23.271
4	−4.4	20.224	23.951
5	−4.4	20.326	23.145
6	−4.3	19.659	22.878
7	−4.3	20.154	23.001
8	−4.3	5.542	8.583
9	−4.2	18.811	23.100
**Binding affinity of hexadecanoic acid, methyl ester**			
1	−4.1	0.000	0.000
2	−3.8	17.548	19.890
3	−3.7	16.591	19.286
4	−3.6	26.541	28.701
5	−3.6	1.712	1.958
6	−3.5	3.957	7.771
7	−3.5	21.723	24.733
8	−3.4	16.952	19.951
9	−3.4	3.886	8.056
**Energies of TXNIP with 9-octadecenoic acid (Z)-, methyl ester**			
1	−4.5	0.000	0.000
2	−4.4	20.879	23.520
3	−4.4	20.350	23.271
4	−4.4	20.224	22.951
5	−4.4	20.326	23.145
6	−4.3	19.659	22.878
7	−4.3	20.154	23.001
8	−4.3	5.542	8.583
9	−4.2	18.811	23.100
**Energies of TXNIP with hexadecanoic acid, methyl ester**			
1	−4.1	0.000	0.000
2	−3.8	17.548	19.890
3	−3.7	16.591	19.286
4	−3.6	26.541	28.701
5	−3.6	1.712	1.958
6	−3.5	3.957	7.771
7	−3.5	21.723	24.733
8	−3.4	16.952	19.951
9	−3.4	3.886	8.056
**Energies of TXNIP with n-hexadecanoic acid**			
1	−4.5	0.000	0.000
2	−4.4	20.003	22.424
3	−4.2	1.604	2.535
4	−4.0	19.607	20.856
5	−4.0	21.531	22.971
6	−4.0	25.287	26.224
7	−4.0	24.777	26.230
8	−3.9	25.253	26.260
9	−3.9	18.834	19.952
**Energies of TXNIP with tetradecane, 2,6,10-trimethyl**			
1	−4.5	0.000	0.000
2	−4.3	22.755	25.716
3	−4.3	2.085	6.502
4	−4.2	22.799	25.683
5	−4.2	17.737	19.172
6	−4.2	1.189	3.400
7	−4.0	17.879	19.791
8	−4.0	1.548	2.634
9	−3.9	17.927	19.445
**Energies of TXNIP with bis (2-ethylhexyl) phthalate**			
1	−5.0	0.000	0.000
2	−4.8	26.261	29.495
3	−4.8	25.759	29.424
4	−4.8	25.607	29.561
5	−4.8	1.108	2.489
6	−4.7	25.485	29.490
7	−4.7	1.350	7.700
8	−4.6	25.134	28.838
9	−4.6	1.739	3.010
**Energies of TXNIP with decane, 3,8-dimethyl**			
1	−4.0	0.000	0.000
2	−3.8	17.829	19.803
3	−3.8	1.674	3.031
4	−3.7	1.720	3.959
5	−3.7	17.763	19.245
6	−3.6	2.831	4.993
7	−3.5	2.533	5.213
8	−3.4	16.354	18.655
9	−3.4	17.728	19.545

#### *In silico* analysis

Docking analysis was performed by making proteins, that is, thioredoxin interaction protein (TXNIP), to interact with different ligands identified from the GC*-*MS analysis of methanol and *n*-hexane extracts. The 3D structure of the protein was retrieved from a protein data bank (PDB).

#### *In silico* docking analysis of methanol-identified compounds

**Figure 12:**
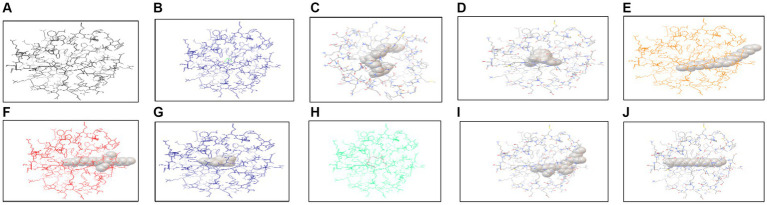
Interaction of TXNIP proteins with different metabolites extracted from *P. granatum* predicted using AutoDock Vina. **(A)** 3,5-octadien-2-one, **(B)** 1H-pyrrole -2,5-dione, **(C)** beta-D-lyxofuranoside, 5-O-(beta-D-lyxofuranosyl)-decyl, **(D)** diethyl phthalate, **(E)** 9-octadecenoic acid (Z)-, methyl ester, **(F)** hexadecanoic acid, methyl ester, **(G)** *n*-hexadecanoic acid, **(H)** tetradecane, 2,6,10-trimethyl, **(I)** bis (2-ethylhexyl) phthalate, and **(J)** decane, 3,8-dimethyl.

**Figure 13:**
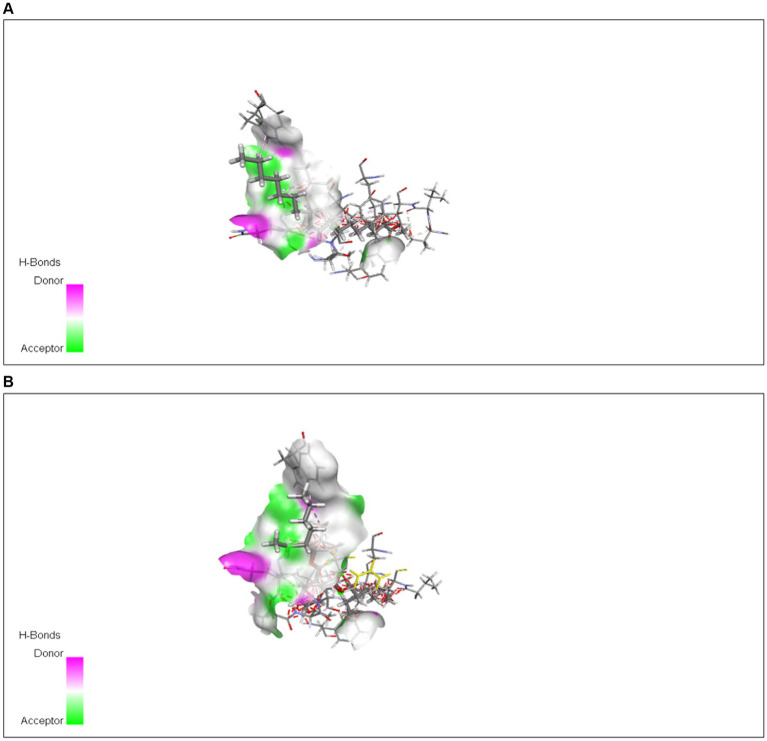
Interaction of **(A)** 9-octadecenoic acid (Z)-, methyl ester with TXNIP from methanol extract and with **(B)** bis (2-ethylhexyl) phthalate from *n*-hexane extract.

*In silico* analysis was performed using AutoDock Vina, which is an offline tool providing the interaction of protein with ligands. Protein and ligand interaction was observed. Docking was performed for the identification of interaction between both of these. Different ligands interacted with protein and few of them showed the highest interaction in the form of energies (Table 6). The docked structure of TXNIP with different ligands is shown in Figures 12, 13.

The interaction of protein was checked through AutoDock Vina using different ligands identified from methanol and *n*-hexane extracts through GC*-*MS. Various interactions were checked out with TXNIP in methanol extract, and the highest interactions were observed by 9-octadecenoic acid (Z)-, methyl ester from methanol and bis (2-ethylhexyl) phthalate from n-hexane extract, with energy values of −4.5 and −5.0 kcal/mol, respectively.

## Discussion

It is well recognized that plants are a source of chemicals for the creation of novel medications. Pomegranate contains approximately 60% peel in its fruit. Punicalins, ellagic acid, punicalagins, gallic acid, and gallotannis are the phytochemicals present in pomegranate peel. The non-edible parts including flowers, barks, peel, leaves, and buds play significant roles in nutritional characteristics in addition to the fruit’s edible part. Compared to the fruit’s edible portion, the peel is usually known as waste but it contains biologically active compounds rich in nutrients, so the peel has more therapeutic benefits. Due to a number of issues, including the emergence of resistance and the adverse effects connected with the use of contemporary medications like antibiotics and anti-cancer medications, there is a huge interest in medicinal plants today (15).

Environmental pollution is caused when by-products such as seeds and peels are produced. A significant number of biological components is present in the peel and it contains approximately 26%–30% of the total weight of fruit. This research provided the health benefits of pomegranate peel, and the identification of biological components like phenolic acids, alkaloids, flavonoids, tannins, minerals, dietary fibers, and vitamins were assessed. We found that the peel had cinnamic, butyric, caffeic, erucic, ellagic, ferulic, chlorogenic, and gallic acids. Environmental conditions affected the concentrations and phenolic acid profiles. Pomegranate peel contains different bioactive components like caffeic (20.56 mg), ellagic (35.89 mg), and gallic (123.79 mg) acids. Dietary fiber was used as a natural source and the peel was rich in dietary fibers of approximately 33% to 62%. Dietary fibers included uronic acid, lignin, sugars, and cellulose, but the maximum amount was present in lignin. Neural sugars like galactose, rabinose, and xylose were present in the peel. Uronic acid and cellulose contained the same amount of approximately 16 to 22 mg/100 mg in pomegranate peel. Peel contained gallic acid, punicalagin, ellagitannins, and ellagic acids, showing antibacterial effects against *E. coli* and *S. aureus* microbes, which inhibited glycosyltransferases that cause cell death. Peel showed inhibitory effects against *Yersinia enterocolitica*, *S. aureus*, *L. monocytogenes*, and *E. coli* which was confirmed by *in vivo* studies (9).

Anti-diabetic activity was done by α-glucosidase assay. Phosphate buffer with 0.07 M molarity and 6.8 pH was prepared with p-NPG substrate and α-glucosidase enzyme (2.0 U m/L and α- glucosidase 2.5 U m/L). Results were measured at 405 nm by ELISA assay. The highest activity was observed in methanol and butanol showed the lowest activity. The greatest activity was exhibited by methanol and butanol extracts. The percentage concentration of extracts provided the highest percentage of methanol extract at 80.5% (29) (25). In the antibacterial assay, *S. viridans* was used as gram +ve and *E. coli* as gram –ve bacterial strains. Extracts of methanol and butanol showed resistance against gram +ve bacteria. Peel extract of methanol and butanol showed antibacterial activity against *S. viridans*. The zone of inhibition was measured in mm of different extracts of *P. granatum* peel. Maximum zones were shown by butanol and methanol extracts at 0.5 mg/mL is 16 ± 0.39 and 15 ± 0.34, respectively (30).

*L. monocytogenes* exhibited the lowest bactericidal effects when consumed with 24.7 mg/mL of peel extracts. In our study, photochemistry screening confirmed the presence of phenols and tannins in methanol and *n*-hexane extract but not in chloroform and butanol fractions. Glycosides, flavonoids, and terpenoids were present in all extracts except *n*-hexane. Tannins were present in all fractions except chloroform. Tannins were present in butanol and *n*-hexane but not in methanol and chloroform. Steroids were absent in butanol and chloroform. Previous studies also reported that saponins, steroids, and flavonoids were present in extracts of *P. granatum.* Our research proved that the antioxidant potential of *P. granatum* and its fractions was determined by using the DPPH radical scavenging assay (35). Radical scavenging activity was evaluated at different concentrations of fractions. Results were analyzed statistically. Methanol, chloroform, *n*-hexane, and butanol showed antioxidant potential at 0.5 mg/mL concentrations of 85 ± 0.096, 71 ± 0.029, 65 ± 0.050, and 89 ± 0.019, respectively. When concentration decreased, antioxidant potential also decreased to 80 ± 0.029, 60 ± 0.025, 58 ± 0.049, and 86.6 ± 0.023 at 0.25 mg/mL for methanol, chloroform, *n*-hexane, and butanol concentration, respectively. Butanol and methanol fractions showed the highest antioxidant activity. The order of antioxidant activity results was butanol > methanol > chloroform > *n*-hexane. Ascorbic acid was taken as standard which showed radical scavenging percentage at different concentrations, i.e., 0.5, 0.25, 0.125, 0.0625, and 0.03125 mg/mL as 98.9 ± 0.007, 98.5 ± 0.005, 97.3 ± 0.012, 96.6 ± 0.015, and 96 ± 0.014, respectively. The results of anti-inflammatory properties showed that fractions at concentrations of 0.5 mg/mL extracts for methanol, chloroform, *n*-hexane, and butanol were 90 ± 0.034, 66 ± 0.017, 77 ± 0.060, and 78 ± 0.017, respectively. When concentration decreased, inhibition values also decreased. At 0.25 mg/mL, inhibition decreased to 86 ± 0.016, 60 ± 0.020, 75 ± 0.045, and 72 ± 0.009, respectively, according to fractions. Dicloran was taken as standard which showed its maximum inhibition of 98 ± 0.005, 97.8 ± 0.015, 96.6 ± 0.018, 95 ± 0.019, and 94 ± 0.012 at different concentrations of 0.5, 0.25, 0.125, 0.625, and 0.03125 mg/mL, so dicloran exhibited inhibition percentages of 98%, 97.8%, 96.6%, 95%, and 94% (28).

Docking of ligands obtained from GC*-*MS was done through AutoDock Vina (36). Compounds that were identified in methanol extract were thymine, 3,5-octadien-2-one, 4H-pyran-4-one, 2,3-dihydro-3,5-dihydroxy-6-methyl, 1H-pyrrole-2,5-dione, diethyl phthalate, benzeneethanamine, N-[(pentafluorophenyl)methylene]-beta.,3,4-tris[(trimethylsilyl)oxy], 2-(2′,4′,4′,6′,6′,8′,8′-heptamethyltetrasiloxan-2′-yloxy)-2,4,4,6,6,8,8,10,10-nonamethylcyclopentasiloxane, 9-octadecenoic acid (Z)-, methyl ester, oxalic acid, 2-ethylhexyl tetradecyl ester, octasiloxane, 1,1,3,3,5,5,7,7,9,9,11,11,13,13,15,15-hexadecamethyl, and more, of which 9-octadecenoic acid (Z)-, methyl ester showed the highest energy and interaction with TXNIP protein. Compounds in *n*-hexane extract were 2,6-Di-tert-butyl-4-hydroxy-4-methylcyclohexa-2,5-dien-1-one, 7,9-Di-tert-butyl-1-oxaspiro(4,5)deca-6,9-diene-2,8-dione, hexadecanoic acid, methyl ester, *n*-hexadecanoic acid, octasiloxane, 1,1,3,3,5,5,7,7,9,9,11,11,13,13,15,15-hexadecamethyl, decane, 3,8-dimethyl, bis (2-ethylhexyl) phthalate, arsenous acid, tris(trimethylsilyl) ester, and others. From all of these compounds, bis (2-ethylhexyl) phthalate showed the highest energy and interaction with protein. So, octadecenoic acid (Z)-, methyl ester and bis (2-ethylhexyl) phthalate can be used for making novel drugs against diabetes. The literature reports various studies documenting phytochemical and pharmacological characterizations of *P. granatum* peel (37–39).

## Conclusion and future perspectives

The study substantiates the value of pomegranate fruit peel waste as an economical source of versatile biotherapeutics. Ongoing studies should unlock additional bioactive secondary metabolites for nutraceutical and pharmaceutical applications via green extraction approaches. Further translational research can facilitate the development of new evidence-based drugs, functional foods, and preventive strategies by leveraging pomegranate peel derivatives against inflammation, diabetes, oxidation, and infection.

## Data availability statement

The mass spectrometry data generated and analyzed in this study have been deposited in a publicly accessible repository. Specifically, the raw mass spectrometry files, processed data files, and associated metadata have been uploaded to the MassIVE repository (https://massive.ucsd.edu), which is a public data repository for mass spectrometry datasets.

## Author contributions

TAs: Methodology, Investigations, Writing – original draft. AAr: Conceptualization, Methodology, Investigations, Supervision, Writing – original draft. SA: Conceptualization, Formal analyses, Supervision, Writing – original draft. FM: Formal analyses, Writing – original draft. KA: Methodology, Investigations, Writing – original draft. UK: Conceptualization, Formal analyses, Writing – original draft. SR: Methodology, Supervision, Writing – original draft. TAf: Methodology, Investigations, Writing – original draft. AAl: Conceptualization, Methodology, Investigations, Supervision, Writing – original draft. HS: Formal analyses, Investigations, Writing – original draft. MOI: Methodology, Investigations, Writing – original draft. MZ: Conceptualization, Formal analyses, Writing – original draft.
